# A Comparison of the Charlson and Elixhauser Methods for Predicting Nursing Indicators in Gastrectomy with Gastric Cancer Patients

**DOI:** 10.3390/healthcare11131830

**Published:** 2023-06-22

**Authors:** Chul-Gyu Kim, Kyun-Seop Bae

**Affiliations:** 1 Department of Nursing, Chungbuk National University, Cheongju 28644, Republic of Korea; cgkim@cbnu.ac.kr; 2 Asan Medical Center, University of Ulsan College of Medicine, Seoul 05505, Republic of Korea

**Keywords:** nursing care, patient outcome assessment, risk adjustment

## Abstract

**Background:** Comorbidity indices such as Charlson’s (CCI) and Elixhauser’s (ECI) are used to adjust the patient’s care, depending on the severity of their condition. However, no study has compared these indices’ ability to predict nursing-sensitive outcomes (NSOs). We compared the performance of CCI and ECI in predicting NSOs in gastric cancer patients’ gastrectomy. **Methods:** Gastric cancer patients with gastrectomy, aged 19 years or older and admitted between 2015 and 2016, were selected from the Korea Insurance Review and Assessment Service database. We examined the relationships between NSOs and CCI or ECI while adjusting patient and hospital characteristics with logistic regression. **Results:** The ECI item model was the best in view of the C-statistic and Akaike Information Criterion for total NSO, physiologic/metabolic derangement, and deep vein thrombosis, while the Charlson item model was the best for upper gastrointestinal tract bleeding. For the C-statistic, the ECI item model was the best for in-hospital mortality, CNS complications, shock/cardiac arrest, urinary tract infection, pulmonary failure, and wound infection, while the CCI item model was the best for hospital-acquired pneumonia and pressure ulcers. **Conclusions:** In predicting 8 of 11 NSOs, the ECI item model outperformed the others. For other NSOs, the best model varies between the ECI item and CCI item model.

## 1. Introduction

With the increase in the size of the aging population, the incidence of cancer is also increasing. Stomach cancer is one of the most frequently occurring cancers in South Korea, and cases of gastric cancer numbered 26,662 in 2020 [1]. The major reasons for the higher number of gastric cancer cases are high-salt food, spicy and charred meat, smoking, drinking, and Helicobacter pylori infection [2]. The main treatment for gastric cancer without metastasis is gastrectomy [3]. The incidence of gastrectomy is the highest among gastrointestinal (GI) operations for cancer in Korea [4].

A reliable measure for surgical quality is always necessary. Direct measures of surgical outcomes are the best for measuring quality [5]. In the same context, measuring nursing outcomes are the best for measuring nursing quality [6]. Nursing-sensitive outcomes (NSOs) serve as valuable tools for monitoring and improving nursing quality. They are indicators of the patient’s health status that result directly from the care provided by nurses [7]. Those for adults include in-hospital death, central nervous system (CNS) complications, shock or cardiac arrest, hospital-acquired pneumonia, upper gastrointestinal tract bleeding, pressure ulcers, deep vein thrombosis, urinary tract infection, wound infection, pulmonary failure, sepsis, and physiologic/metabolic derangement [8,9]. Nonetheless, it is necessary to adjust these indicators based on patient acuity to effectively compare variations in nursing quality among different hospitals [10]. 

The quality and safety of healthcare, including nursing care, is becoming more and more important, despite of finite resources. In accordance with this, skill-mixing and nursing modes need to be redesigned. In this regard, more research is focused on addressing the impact of nursing components such as nurse staffing, nurse characteristics, clinical nursing support, and the nursing practice environment on the quality and safety of patient care [11]. The impact of hospital structures and the processes of nursing care on outcomes can be examined with adjustments in patient factors [12]. Therefore, an appropriate risk adjustment model is essential in nursing quality studies [5]. Administrative databases are large, and they are easy to access for research into care quality after multiple surgical procedures and nursing care [13,14,15,16]. 

Comorbidity indices such as Charlson’s (CCI) and Elixhauser’s (ECI) from an administrative database are widely used to adjust the patient’s risk level in health services research [17]. In 1987, Charlson et al. developed a method for patient risk adjustment using administrative data, which includes 19 clinical diagnoses (due to their relevance to 1-year mortality). The index is the sum of the weighted score for these diagnoses. Several studies have validated this index [18,19,20]; since then, it has been the one most widely used to assess comorbidity. After publication of ICD-10 codes, the Charlson’s method was also translated from ICD-9 to ICD-10 codes [21].

Another popular method for patients’ risk adjustment is that of Elixhauser et al., created in 1998 [22]. It includes 30 comorbidity measures, so is more comprehensive. The Elixhauser comorbidity method can be used to predict in-hospital mortality, hospitalization length, and hospital charges. Complications during hospitalization should be distinguished from pre-existing diagnoses before admission when using this index.

There are several studies that compare the performance of the Charlson and Elixhauser methods in various population [23,24,25,26,27,28]. The Elixhauser method seems better than the Charlson method for risk adjustment because it has a higher C-statistic than the Charlson method. Lieffers et al. [23] studied these methods with colorectal cancer patients for survival prediction, Quail et al. [24] with a general population, diabetic patients, and osteoporosis patients for mortality and hospitalization prediction, and Chang et al. [25] and Lee et al. [26] for oral cancer survival. Tsai et al. [27] showed that in patients with schizophrenic disorder, for mortality prediction, ECI was superior, and Baron et al. [28] reported that ECI was better than CCI in predicting minor complications, airway complications, hemorrhagic anemia, myocardial infarction, cardiac arrest, urinary tract infection, and pulmonary embolism after anterior lumbar interbody fusion operations. 

However, no study has compared these indices’ ability to predict nursing-sensitive outcomes (NSOs) in patients with gastric resections. Consequently, it is not known how accurately these methods can adjust for NSOs in patients with gastric resections. We used the Korean Health Insurance Review and Assessment Service (Korea HIRA) database to investigate the incidences of comorbid conditions, and to compare the predictive performance of CCI and ECI for the NSOs of gastric cancer patients with gastrectomy.

### Objective

The objective of this study was to compare the performance of two common indices in predicting the NSOs of patients with gastric resections for gastric cancer.

The more specific objectives are

1. To identify incidences of comorbid conditions using the Korean HIRA reimbursement data of gastric resection patients with gastric cancer;
2. To compare the predictive performance of CCI and ECI for the NSOs of gastric cancer patients with gastrectomy.

## 2. Materials and Methods

The methodology employed in this study is akin to the authors’ prior research that utilized a database from the identical institution [29]. However, the aim and the operative procedure under consideration—a comparison between the Charlson and Elixhauser indices concerning gastrectomy for primary gastric cancer—differ from the authors’ previous investigation.

### 2.1. Database

We utilized the Korea HIRA database, encompassing reimbursement claims for inpatient and outpatient services from 2015 to 2016. All hospitals in Korea, including tertiary and general ones, must submit the reimbursement claims of their patients to HIRA electronically. This database contains patient demographics (sex and age), diagnoses up to 30, all types of operations and procedures provided, and the details of patient care (including the usage of an emergency room or intensive care unit). Diagnoses are coded by the International Classification of Diseases, tenth revision (ICD-10), while procedure codes are from the Korean claim code of HIRA.

### 2.2. Participants

#### 2.2.1. Inclusion Criteria

Using electronic reimbursement claims data, we identified all patients aged 19 years or older who had undergone gastrectomy for gastric cancer (identified by ICD-10 code C16, which refers to malignant neoplasm of the stomach) between January 2015 and December 2016. The patient information we used was anonymized. 

#### 2.2.2. Exclusion Criteria

Patients without nationwide individual identification numbers were excluded from the analysis, as their previous inpatient and outpatient claim data could not be tracked, and their comorbidity scores (measured by Charlson’s and Elixhauser’s comorbidity indices) could not be calculated. In addition, only primary cancers were included in the analysis, while metastatic cancers and those originating from adjacent organs, such as the colon, pancreas, or gallbladder, were excluded.

### 2.3. Patient and Hospital Characteristics 

The patient characteristics used in the analysis were sex, age, and medical security type. The medical security type can be used as an indicator of socioeconomic status (SES); health insurance represents a relatively higher SES, and medical aid represents a lower SES.

The characteristics of clinical practice were the route of admission, the use of ICU, the type of gastrectomy (subtotal or total), and lymph node dissection. Route of admission is a measure of relative urgency and is categorized as outpatient or emergency room.

The hospital characteristics included hospital location and type. The hospital location was categorized as Seoul (the capital of Republic of Korea), metropolitan, and city. The hospital type was classified as tertiary hospital, general hospital, or hospital.

### 2.4. Measures 

#### 2.4.1. Comorbidity Methods 

We compared the two comorbidity indices, Charlson (CCI) and Elixhauser (ECI). The Charlson method includes 17 diagnostic categories, while the Elixhauser method includes 30 diagnostic categories. Comorbidities for the Charlson’s and Elixhauser’s indices were identified by the ICD-10 code. Each patient’s comorbidities were identified by the ICD-10 code within six months before admission, using outpatient and inpatient service records. However, newly diagnosed comorbidities identified during the gastrectomy admission were not included, because those cannot be distinguished from the complications of inpatient treatment. Therefore, CCI and ECI were calculated with the identified pre-existing comorbidities at the time of hospitalization for the gastrectomy.

#### 2.4.2. Nursing-Sensitive Outcomes

Eleven NSOs were selected, including in-hospital death, CNS complications, shock/cardiac arrest, hospital-acquired pneumonia, upper GI tract bleeding, pressure ulcers, deep vein thrombosis, urinary tract infection, wound infection, pulmonary failure, and physiologic/metabolic derangement. The definition of each NSO was based on Needleman et al. [30], who used ICD-9 codes, and Wilson et al. [7], who used ICD-10 codes and Australian DRG. ICD-10 codes and Korean diagnosis-related groups (DRG) to match each NSO were determined with the consultation of HIRA’s medical recorder and researchers. The dividend, divisor, and exclusion criteria for each NSO were defined as the above, and the 11 NSO incidences were calculated using HIRA’s inpatient reimbursement data.

### 2.5. Statistical Analysis

Data analysis was performed using SAS^®^ 9.4 (Cary, NC, USA) software. Descriptive statistics, including mean, standard deviation (SD), frequency, and proportion, were used to summarize patient and hospital characteristics and NSO status. The admission case was considered the unit of elementary event. The differences in proportions and means by patient and hospital characteristics were tested using the chi-squared test and Student’s *t*-test. Univariate and multiple logistic regression were used to examine the relationship between CCI or ECI and NSOs, adjusted with patient and hospital characteristics. The base model included admission route (outpatient or emergency room as an urgency indicator), gastrectomy type (total or subtotal), lymph node dissection, hospital location, and hospital type. Item models are models in which CCI or ECI disease items are added to the base model as independent variables. Continuous models are models in which CCI or ECI scores are added as quantitative independent variables. Categorical models are models in which categorized CCI (Charlson’s score <3 or ≥3) or ECI (Elixhauser’s score < 0, 0, 1–4, ≥5) are added to the base model. Two-sided tests were used for all statistical tests, and statistical significance was deemed with *p* < 0.05. The Akaike information criterion (AIC) and C-statistic were the indicators used to assess the performance and discriminative ability of the methods over the base model, which includes sex, age, medical security type, admission route, gastrectomy type, lymph node dissection, hospital location, and hospital type. The AIC was calculated as 2k–2ln(L), where k is the number of parameters of the model, and L is the maximum likelihood. A smaller AIC value corresponds with the better data explanation ability of the model. The C-statistic shows the discrimination ability, with values of 0.5 (no ability), 0.7 to 0.8 (acceptable), 0.8 to 0.9 (excellent), 0.9 to 1.0 (outstanding), and 1 (perfect discrimination).

## 3. Results

### 3.1. Patient and Hospital Characteristics

The study included 33,323 inpatient cases of gastrectomy for gastric cancer in Korea between 2015 and 2016, among which 3545 (10.64%) had at least one NSO indicator. Patients with at least one NSO were significantly different from those without any NSO indicator in terms of sex, age, medical security type, admission route, ICU experience, gastrectomy type, hospital location, hospital type, Charlson’s comorbidity score, and Elixhauser’s comorbidity score (*p* < 0.001). Patients with at least one NSO had a longer average length of hospital stay (27.92 days) compared to those without any NSOs (23.23 days) (*p* < 0.001). Lymph node dissection did not show a significant difference in NSO rate (*p* = 0.247; Table 1).

The mean age of patients with at least one NSO indicator was 65.99 years, which was higher than that of patients without any NSO indicator (61.46 years) (*p* < 0.0001). Males had a higher incidence rate of NSO (11.33%) compared to females (9.25%) (*p* < 0.001). Patients with medical aid had a higher NSO rate (19.51%) than patients with health insurance (*p* < 0.001). The NSO rate was higher for patients admitted via emergency room than those admitted via outpatient department (*p* < 0.001). Patients who underwent total gastrectomy had a higher NSO rate than those who underwent subtotal gastrectomy (*p* < 0.001). The NSO rate was higher in patients who had experienced ICU care and had higher Charlson’s or Elixhauser’s comorbidity scores (*p* < 0.001). Patients admitted to hospitals in large metropolitan areas or tertiary hospitals had a lower NSO rate compared to those admitted to hospitals in city areas or non-tertiary hospitals (*p* < 0.001).

### 3.2. Charlson and Elixhauser Comorbidities in Patients

The comorbidity rates of peptic ulcer, diabetes mellitus and hypertension were more than 5% in patients with gastrectomy. Comorbidities such as AIDS/HIV, lymphoma, obesity, and drug abuse were less than 0.1%.

Patients with any NSO indicator had significantly different Charlson’s comorbidities than their counterparts, including myocardial infarction, congestive heart failure, peripheral vascular disease, cerebrovascular disease, dementia, chronic pulmonary disease, rheumatic disease, peptic ulcer disease, mild and moderate liver disease, diabetes mellitus without and with end-organ damage, hemiplegia, renal disease, any malignancy, and metastatic solid tumor (*p* < 0.05). There was no significant difference in NSO rate by AIDS/HIV (*p* = 0.7301). 

Patients with any NSO indicator had significantly different Elixhauser’s comorbidities than their counterparts, including congestive heart failure, cardiac arrhythmias, valvular disease, pulmonary circulation disorders, peripheral vascular disorders, hypertension, paralysis, neurodegenerative disorders, chronic pulmonary disease, diabetes mellitus both uncomplicated and complicated, hypothyroidism, renal failure, liver disease, peptic ulcer disease excluding bleeding, metastatic cancer, solid tumor without metastasis, rheumatoid arthritis/collagen vascular diseases, coagulopathy, weight loss, fluid and electrolyte disorders, blood loss anemia, deficiency anemia, alcohol abuse, drug abuse, psychoses, and depression (*p* < 0.05). There was no significant difference in NSO rate by AIDS/HIV, lymphoma, and obesity (*p* > 0.05; Table 2).

### 3.3. Adjusted Odds Ratio of Nursing Indicators Using Charlson and Elixhauser Comorbidities 

Table 3 shows the non-adjusted and adjusted odds ratios for the NSO indicators. Congestive heart failure, myocardial infarction, cerebrovascular disease, hemiplegia, dementia, peripheral vascular disease, rheumatic disease, chronic pulmonary disease, mild and moderate liver disease, peptic ulcer disease, diabetes mellitus with or without end-organ damage, renal disease, metastatic solid tumor, and any malignancy in Charlson’s comorbidities were significant factors after adjustment with patient- and hospital-related characteristics (*p* < 0.05). Those diseases significantly increased the risk of NSO incidence. Additionally, Charlson’s comorbidity score (≥3) had an odds ratio of 1.99, compared to the Charlson’s comorbidity score, which was less than 3 (Table 3).

Elixhauser’s comorbidities with the adjustment of control variables were significant factors for cardiac arrhythmias, congestive heart failure, paralysis, hypertension, pulmonary circulation disorders, chronic pulmonary disease, peripheral vascular disorders, neurodegenerative disorders, renal failure, diabetes mellitus both uncomplicated and complicated, peptic ulcer disease without bleeding, hypothyroidism, liver disease, solid tumor without metastasis, metastatic cancer, rheumatoid arthritis/collagen vascular diseases, weight loss, coagulopathy, fluid and electrolyte disorders, alcohol abuse, deficiency anemia, depression, and psychoses (*p* < 0.05). Those diseases significantly increased the risk of NSO incidence. However, valvular disease, blood loss anemia, obesity, lymphoma, and drug abuse were not significant factors (*p* > 0.05). Additionally, Elixhauser’s comorbidity score (0) had an odds ratio of 0.52, compared to the Elixhauser’s comorbidity score, which was less than 0 (Table 3).

### 3.4. Comparison of Charlson and Elixhauser Comorbidities with Respect to Nursing Indicator

**Figure 1:**
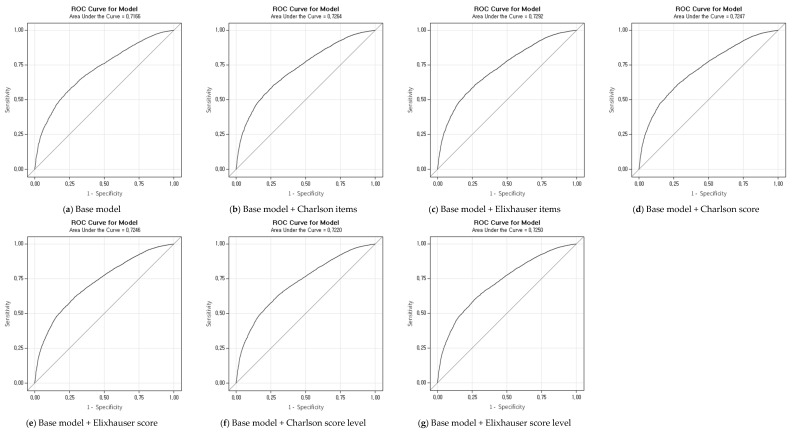
ROC curve of nursing-sensitive outcomes by model.

Table 4 shows the AIC and C-statistics for the Charlson and Elixhauser comorbidity methods adjusted with sex, age, medical security type, admission route, gastrectomy type, lymph node dissection, hospital location, and hospital type. The Elixhauser item model was the best comorbidity risk adjustment method with the lowest AIC, highest C-statistic and receiver operating characteristic (ROC) curve for total NSOs (AIC 20518.49, C-statistics 0.729, Figure 1), deep vein thrombosis (AIC 2539.31, C-statistics 0.741), and physiologic/metabolic derangement (AIC 9405.67, C-statistics 0.733). The Charlson item model was the best comorbidity risk adjustment method with the lowest AIC and highest C-statistic for upper GI tract bleeding (AIC 4753.04, C-statistics 0.793). The Elixhauser item model was the best comorbidity risk adjustment method with the highest C-statistic for in-hospital mortality (C-statistics 0.863), CNS complications (C-statistics 0.804), shock/cardiac arrest (C-statistics 0.779), urinary tract infection (C-statistics 0.746), pulmonary failure (C-statistics 0.785), and wound infection (C-statistics 0.799). The Charlson item model was the best comorbidity risk adjustment method with the highest C-statistic for hospital-acquired pneumonia (C-statistics 0.747) and pressure ulcers (C-statistics 0.759). When the comorbidities were summed as a weighted single score, the Elixhauser item model performed better than the Charlson item model (Table 4).

As the emphasis on improving surgical and nursing quality intensifies, evaluating the preoperative risks of surgical candidates remains crucial. The patient’s particular comorbidity burden is one of the most crucial factors to consider during this assessment. While individual comorbidities have been linked to an increased risk of several postoperative adverse events such as NSOs, the CCI or the ECI is typically used to assess a patient’s comorbidity burden [31]. Despite being frequently used, there is no literature available on whether these two measures can predict NSOs in gastrectomy with gastric cancer. 

We were able to validate each of the two methods for this patient population, as overall C-statistics were more than 0.72. A C-statistic approximating 0.75 is considered acceptable for discrimination, and validates methods for ongoing use [16]. This result showed the excellent performance of the model for in-hospital mortality and CNS complications, with C-statistics of 0.80–0.90, and acceptable performance regarding other NSOs, with C-statistics of 0.70–0.80 [28]. However, Baron et al. [28] showed the poor performance (C-statistics < 0.70) of CCI for cardiac arrest, pneumonia, and surgical site infection, as well as age, sex, race, and primary payer, not including clinical and hospital characteristics such as route of admission, the use of intensive care unit, type of gastrectomy, hospital location, and hospital type. We used these clinical and hospital characteristics in addition to ECI or CCI, so the C-statistics exceeded 0.70. Meanwhile, the C-statistic was 0.695 in deep vein thrombosis without ECI or CCI. Therefore, it is necessary to use ECI or CCI to accurately predict NSOs in addition to clinical and hospital characteristics, since the clinical and hospital characteristics were associated with occurrence of NSO. 

This study found the Elixhauser model was a better predictor of total NSOs, and eight of the eleven NSOs, than the CCI model. This study found the ECI statistically superior to the CCI in predicting in-hospital mortality, CNS complications, shock/cardiac arrest, deep vein thrombosis, physiologic/metabolic derangement, urinary tract infection, pulmonary failure, and wound infection during the inpatient postoperative period. In other words, the ECI model also predicted infection-related complications more effectively. The reason for this may be the inclusion of compromised immune function in the ECI, but not in the CCI [28]. Meanwhile, the CCI model significantly outperformed the ECI in the prediction of three of these NSOs: hospital-acquired pneumonia, upper GI tract bleeding, and pressure ulcers. Additionally, models using Elixhauser and Charlson items were better at risk adjustment than models with CCI or ECI as a continuous or categorized variable. 

These results are similar to those of previous studies on anterior cervical discectomy and fusion by Ranson et al. [31]. The studies in patients with anterior cervical discectomy and fusion showed that the CCI model was a poor predictor of any complication after operation, and the ECI model was an excellent method for predicting deep vein thrombosis, in-hospital mortality, wound infection, septic shock, and pulmonary embolism (compared to CCI). Meanwhile, the CCI model was better in the prediction of hospital-acquired pneumonia than ECI [31]. Baron et al. [28] reported that the ECI model was a better method than CCI for predicting cardiac arrest, pulmonary embolism, and wound infection. Grendar et al. [16] showed that the CCI model was a poor predictor of in-hospital mortality in gastric resection. In addition, the CCI model was an acceptable predictor of any major complication, but a poor predictor of any minor complication. The Elixhauser model was slightly better, producing acceptable predictions of major complications, but poor predictions of minor complications. These results suggest that both models have some values in predicting postoperative complications [31]. However, the selection of the adjustment model could vary between the ECI item and CCI item models for specific nursing indicators, based on the predictive performance. Therefore, it is better to use the ECI item model for predicting in-hospital mortality, CNS complications, shock/cardiac arrest, deep vein thrombosis, physiologic/metabolic derangement, urinary tract infection, pulmonary failure, and wound infection, and to use the CCI item model for predicting hospital-acquired pneumonia, upper GI tract bleeding, and pressure ulcers in inpatients with gastrectomy. This multiple logistic model may be incorporated into electronic nursing recording (ENR), and automatically warn of the risk of NSO.

Meanwhile, the C-statistics of CCI and ECI models for in-hospital mortality were the highest, at 0.853 and 0.863, respectively. These results reflect that CCI and ECI were developed to predict 1-year mortality and in-hospital mortality. To improve the prediction power of each indicator, we need to develop an adjustment method by indicator, such as the Braden scale, or the surgical pressure ulcer risk scale for pressure ulcers [32,33]. We could not adjust using Braden scale for pressure ulcers, since the measurement of the Braden scale was not included in the reimbursement data, although a nurse may have measured the Braden scale of a given patient at the time of their hospital admission [34]. To measure nursing quality effectively, the policy should include the Braden scale or a score of surgical site infection [35] in the administrative data.

The superior performance of the ECI can likely be attributed to its better characterization of the underlying comorbidity burden, achieved by adding more comorbidities not included in the CCI [36]. Some of the patient factors included in the ECI but excluded from the CCI have been identified as strong predictors of NSOs for gastric resection, such as pulmonary circulation disorders, fluid and electrolyte disorders, and coagulopathy. The range of odds ratios of comorbidities in CCI was from 1.12 (metastatic solid tumor) to 2.67 (congestive heart failure). However, the range of odds ratios of comorbidities in ECI was from 1.31 (peptic ulcer disease excluding bleeding) to 10.15 (pulmonary circulation disorder). There are five comorbidities in ECI with odds ratios more than 2.67: pulmonary circulation disorder, paralysis, neurodegenerative disorders, coagulopathy, and fluid and electrolyte disorders. Patients with severe coagulopathy and fluid and electrolyte disorders [16] undergoing gastric resection are at greater risk of in-hospital mortality. Therefore, nurses should frequently monitor patients’ conditions and provide nursing care to prevent the occurrence of NSOs in inpatients with pulmonary circulation disorder, paralysis, neurodegenerative disorders, coagulopathy, and fluid and electrolyte disorders.

In this study, the ORs of AIDS/HIV and drug abuse could not be obtained because there were so few patients with AIDS/HIV (n = 1), and drug abuse (n = 2). The prevalence of AIDS/HIV and drug abuse was very low in Korea. In 2021, in Korea, the total number of patients with AIDS/HIV was 15,196 [37], and the number of patients with drug abuse was 12,613 [38]. It is necessary to select comorbidities by considering their prevalence in the country concerned. A significant association between four Elixhauser-listed comorbidities (vascular disease, lymphoma, obesity, blood loss anemia) and nursing-sensitive outcomes was not found. The prevalence of comorbidities for patients with gastrectomy in this study was from 0% (1 case) to 8.78%. They do not seem to be significant for those diseases with a low volume of patients, such as lymphoma (n = 24) and obesity (n = 22). The obesity prevalence in our study was only 0.07%, which was much lower than the 38.3% in the Korean adult population in 2020 [39]. Our dataset was obtained from the reimbursement database of HIRA in the years 2015~2016. There is a possibility the comorbidity rate is different at the current time, but most hospitals in Korea did not report obesity in the insurance claim if the patients were not treated for obesity at the time of admission. However, patients at the extremes of BMI (underweight or morbidly obese) seem to have the highest postoperative morbidity and mortality risk [40]. It is necessary to record BMI in administrative databases to screen patients at a high risk of developing complications.

Several potential limitations must be considered when interpreting the results of this study. Firstly, large administrative databases may underreport chronic medical conditions. Secondly, the use of ICD-10 codes to identify patient comorbidities may result in coding errors that cannot be fully eliminated. Additionally, as a retrospective study, it may be subject to biases and confounding factors. Moreover, the CCI and ECI models used in this study may not be generalizable to populations receiving different treatments.

## 4. Conclusions

The ECI was found to be more effective than the CCI in predicting eight out of eleven NSOs. Since accurate prediction and prevention of NSOs can enhance patient outcomes and nursing quality, using the ECI (with an item model) to forecast inpatient complications after elective gastrectomy should be taken into consideration. Identifying patients at high risk of developing complications in hospital in the early stages may facilitate the development of intervention strategies to reduce modifiable risk factors. Additionally, the methods demonstrated in this study are utilized by HIRA and other investigators to monitor levels of nursing quality. This monitoring process aims to improve nursing quality and provide patients with information regarding the quality of nursing care in Korean hospitals. Subsequent research should evaluate the performance of ECI in predicting different types of NSOs arising from various operations.

## Figures and Tables

**Table 1:** Distribution of hospital and patient characteristics.

Variables	Total (n = 33,323)	No NSO (n = 29,778)	Any NSO (n = 3545)	*p* Value
Mean Age, y (SD)	61.94 (12.00)	61.46 (11.91)	65.99 (11.95)	<0.0001
<40	1163 (3.49)	1099 (94.50)	64 (5.50)	<0.0001
40–49	4095 (12.29)	3828 (93.48)	267 (6.52)	
50–59	8552 (25.66)	7834 (91.60)	718 (8.40)	
60–69	9566 (28.71)	8597 (89.87)	969 (10.13)	
Over 70	9947 (29.85)	8420 (84.65)	1527 (15.35)	
Sex				
Male	22,284 (66.87)	19,760 (88.67)	2524 (11.33)	<0.0001
Female	11,039 (33.13)	10,018 (90.75)	1021 (9.25)	
Type of medical security				
Health insurance	31,806 (95.45)	28,557 (89.78)	3249 (10.22)	<0.0001
Medical aid	1517 (4.55)	1221 (80.49)	296 (19.51)	
Admission				
Emergency room	893 (2.68)	548 (61.37)	345 (38.63)	<0.0001
Outpatient department	32,430 (97.32)	29,230 (90.13)	3200 (9.87)	
Intensive care unit				
Yes	2694 (8.08)	2237 (83.04)	457 (16.96)	<0.0001
No	30,629 (91.92)	27,541 (89.92)	3088 (10.08)	
OP type				
Subtotal gastrectomy	25,938 (77.84)	23,344 (90.00)	2594 (10.00)	<0.0001
Total gastrectomy	7385 (22.16)	6434 (87.12)	951 (12.88)	
Lymph node dissection				
Yes	32,577 (97.76)	29,121 (89.39)	3456 (10.61)	0.247
No	746 (2.24)	657 (88.07)	89 (11.93)	
Hospital location				
Large metropolitan area (%)	14,365 (43.11)	13,414 (93.38)	951 (6.62)	<0.0001
Other metropolitan area (%)	7685 (23.06)	6872 (89.42)	813 (10.58)	
City area	11,273 (33.83)	9492 (84.20)	1781 (15.80)	
Hospital type				
Tertiary hospital	24,588 (73.79)	22,711 (92.37)	1877 (7.63)	<0.0001
General hospital	8602 (25.81)	6964 (80.96)	1638 (19.04)	
Hospital	133 (0.40)	103 (77.44)	30 (22.56)	
Charlson’s score				
<3	31,545 (94.66)	28,375 (89.95)	3170 (10.05)	<0.0001
≥3	1778 (5.34)	1404 (78.91)	375 (21.09)	
Mean ± SD	0.40 (1.21)	0.36 (1.13)	0.74 (1.71)	<0.0001
Elixhauser’s score				
<0	373 (1.12)	311 (83.38)	62 (16.62)	<0.0001
0	29,485 (88.48)	26,671 (90.46)	2814 (9.54)	
1–4	896 (2.69)	740 (82.59)	156 (17.41)	
≥5	2569 (7.71)	2056 (80.03)	513 (19.97)	
Mean ± SD	1.00 (3.65)	0.88 (3.39)	2.01 (5.26)	<0.0001
Length of stay, day (SD)	23.74(10.39)	23.23 (9.78)	27.92 (13.83)	<0.0001

**Table 2:** Distribution of Charlson and Elixhauser comorbidities in patients.

Comorbidity Scoring Methods	N (%) (n = 33,323)	No NSO (n, %) (n = 29,778)	Any NSO (n, %) (n = 3545)	*p* Value
**Charlson Comorbidities**				
Myocardial infarction	125 (0.38)	100 (80.00)	25 (20.00)	0.0007
Congestive heart failure	276 (0.83)	192 (69.57)	84 (30.43)	<0.0001
Peripheral vascular disease	190 (0.57)	149 (78.42)	41 (21.58)	<0.0001
Cerebrovascular disease	531 (1.59)	408 (76.84)	123 (23.16)	<0.0001
Dementia	132 (0.40)	90 (68.18)	42 (31.82)	<0.0001
Chronic pulmonary disease	698 (2.09)	537 (76.93)	161 (23.07)	<0.0001
Rheumatic disease	63 (0.19)	49 (77.78)	14 (22.22)	0.0028
Peptic ulcer disease	2925 (8.78)	2485 (84.96)	440 (15.04)	<0.0001
Mild liver disease	1255 (3.770	1016 (80.96)	239 (19.04)	<0.0001
Diabetes mellitus without end-organ damage	1947 (5.84)	1556 (79.92)	391 (20.08)	<0.0001
Diabetes mellitus with end-organ damage	271 (0.81)	197 (72.69)	74 (27.31)	<0.0001
Hemiplegia	71 (0.21)	51 (71.83)	20 (28.17)	<0.0001
Renal disease	202 (0.61)	148 (73.27)	54 (26.73)	<0.0001
Any malignancy	918 (2.75)	751 (81.81)	167 (18.19)	<0.0001
Moderate liver disease	50 (0.15)	40 (80.00)	10 (20.00)	0.0317
Metastatic solid tumor	366 (1.10)	297 (81.15)	69 (18.85)	<0.0001
AIDS/HIV	1 (0.00)	1 (100.00)	0 (0.00)	0.7301
**Elixhauser Comorbidities**				
Congestive heart failure	276 (0.83)	192 (69.57)	84 (30.43)	<0.0001
Cardiac arrhythmias	302 (0.91)	231 (76.49)	71 (23.51)	<0.0001
Valvular disease	57 (0.17)	45 (78.95)	12 (21.05)	0.0107
Pulmonary circulation disorders	48 (0.14)	20 (41.67)	28 (58.33)	<0.0001
Peripheral vascular disorders	190 (0.57)	149 (78.42)	41 (21.58)	<0.0001
Hypertension	2141 (6.42)	1745 (81.50)	396 (18.50)	<0.0001
Paralysis	71 (0.21)	51 (71.83)	20 (28.17)	<0.0001
Neurodegenerative disorders	156 (0.47)	113 (72.44)	43 (27.56)	<0.0001
Chronic pulmonary disease	698 (2.09)	537 (76.93)	161 (23.07)	<0.0001
Diabetes, uncomplicated	1727 (5.18)	1395 (80.78)	332 (19.22)	<0.0001
Diabetes, complicated	523 (1.57)	383 (73.23)	140 (26.77)	<0.0001
Hypothyroidism	111 (0.33)	90 (81.08)	21 (18.92)	0.0046
Renal failure	201 (0.60)	147 (73.13)	54 (26.87)	<0.0001
Liver disease	1268 (3.81)	1024 (80.76)	244 (19.24)	<0.0001
Peptic ulcer disease excluding bleeding	1546 (4.64)	1328 (85.90)	218 (14.10)	<0.0001
AIDS/HIV	1 (0.00)	1 (100.00)	0 (0.00)	0.7301
Lymphoma	24 (0.07)	20 (83.33)	4 (16.67)	0.3380
Metastatic cancer	366 (1.10)	297 (81.15)	69 (18.85)	<0.0001
Solid tumor without metastasis	896 (2.69)	733 (81.81)	163 (18.19)	<0.0001
Rheumatoid arthritis/collagen vascular diseases	72 (0.22)	56 (77.78)	16 (22.22)	0.0014
Coagulopathy	129 (0.39)	83 (64.34)	46 (35.66)	<0.0001
Obesity	22 (0.07)	18 (81.82)	4 (18.18)	0.2510
Weight loss	493 (1.48)	397 (80.53)	96 (19.47)	<0.0001
Fluid and electrolyte disorders	398 (1.19)	284 (71.36)	114 (28.64)	<0.0001
Blood loss anemia	73 (0.22)	58 (79.45)	15 (20.55)	0.0060
Deficiency anemia	756 (2.27)	576 (76.19)	180 (23.81)	<0.0001
Alcohol abuse	171 (0.51)	140 (81.87)	31 (18.13)	0.0014
Drug abuse	2 (0.01)	0 (0.0)	2 (100.00)	0.0113 *
Psychoses	52 (0.16)	36 (69.23)	16 (30.77)	<0.0001
Depression	278 (0.83)	209 (75.18)	69 (24.82)	<0.0001

* Fisher’s exact test.

**Table 3:** Adjusted odds ratio of nursing indicators using Charlson and Elixhauser comorbidities.

Comorbidity Scoring Methods	Univariate Analysis OR (95% CI)	Multivariate Analysis OR (95% CI)
**Charlson Comorbidities**		
Myocardial infarction	2.10 (1.35–3.27)	1.67 (1.05–2.65)
Congestive heart failure	3.74 (2.88–4.84)	2.67 (2.03–3.52)
Peripheral vascular disease	2.32 (1.64–3.29)	1.68 (1.17–2.41)
Cerebrovascular disease	2.58 (2.10–3.17)	1.92 (1.55–2.38)
Dementia	3.95 (2.73–5.71)	2.31 (1.56–3.42)
Chronic pulmonary disease	2.59 (2.16–3.10)	1.88 (1.55–2.27)
Rheumatic disease	2.40 (1.32–4.36)	2.37 (1.26–4.46)
Peptic ulcer disease	1.55 (1.39–1.73)	1.44 (1.29–1.62)
Mild liver disease	2.04 (1.77–2.36)	1.80 (1.54–2.09)
Diabetes mellitus without end-organ damage	2.24 (2.00–2.52)	1.87 (1.65–2.12)
Diabetes mellitus with end-organ damage	1.79 (1.56–2.04)	1.60 (1.39–1.85)
Hemiplegia	1.81 (1.40–2.35)	1.64 (1.25–2.14)
Renal disease	1.76 (1.50–2.06)	1.63 (1.38–1.92)
Any malignancy	1.38 (1.27–1.50)	1.34 (1.22–1.46)
Moderate liver disease	1.28 (1.01–1.61)	1.34 (1.06–1.71)
Metastatic solid tumor	1.12 (1.07–1.17)	1.12 (1.07–1.17)
AIDS/HIV	0.30 (NA)	0.29 (NA)
Charlson’s score		
<3	1.00	1.00
≥3	2.39 (2.12–2.69)	1.99 (1.76–2.26)
**Elixhauser Comorbidities**		
Congestive heart failure	3.74 (2.88–4.84)	2.67 (2.03–3.52)
Cardiac arrhythmias	2.61 (2.00–3.42)	1.86 (1.40–2.47)
Valvular disease	2.24 (1.18–4.24)	1.86 (0.96–3.60)
Pulmonary circulation disorders	11.82 (6.65–21.00)	10.15 (5.54–18.59)
Peripheral vascular disorders	2.32 (1.64–3.29)	1.68 (1.17–2.41)
Hypertension	2.02 (1.80–2.26)	1.66 (1.47–1.88)
Paralysis	3.31 (1.97–5.55)	2.69 (1.57–4.62)
Neurodegenerative disorders	3.22 (2.27–4.59)	2.72 (1.88–3.94)
Chronic pulmonary disease	2.59 (2.16–3.10)	1.88 (1.55–2.27)
Diabetes, uncomplicated	2.10 (1.85–2.38)	1.76 (1.54–2.00)
Diabetes, complicated	3.15 (2.59–3.84)	2.52 (2.05–3.11)
Hypothyroidism	1.96 (1.22–3.16)	1.88 (1.14–3.09)
Renal failure	3.12 (2.28–4.27)	2.66 (1.91–3.71)
Liver disease	2.07 (1.79–2.39)	1.83 (1.57–2.13)
Peptic ulcer disease excluding bleeding	1.40 (1.21–1.62)	1.31 (1.13–1.53)
AIDS/HIV	NA (NA)	NA (NA)
Lymphoma	1.68 (0.57–4.93)	2.38 (0.79–7.20)
Metastatic cancer	1.97 (1.51–2.56)	1.98 (1.50–2.61)
Solid tumor without metastasis	1.91 (1.60–2.27)	1.77 (1.48–2.13)
Rheumatoid arthritis/collagen vascular diseases	2.40 (1.38–4.20)	2.25 (1.24–4.11)
Coagulopathy	4.70 (3.27–6.75)	4.43 (3.03–6.49)
Obesity	1.86 (0.63–5.52)	1.36 (0.44–4.17)
Weight loss	2.06 (1.64–2.58)	1.73 (1.37–2.20)
Fluid and electrolyte disorders	3.45 (2.77–4.30)	2.70 (2.14–3.40)
Blood loss anemia	2.17 (1.23–3.84)	1.81 (0.99–3.29)
Deficiency anemia	2.71 (2.28–3.22)	2.07 (1.72–2.49)
Alcohol abuse	1.86 (1.26–2.76)	1.74 (1.15–2.61)
Drug abuse	NA (NA)	NA (NA)
Psychoses	3.74 (2.07–6.76)	2.20 (1.16–4.15)
Depression	2.81 (2.12–3.67)	2.01 (1.50–2.70)
Elixhauser’s score		
<0	1.00	1.00
0	0.52 (0.40–0.70)	0.66 (0.49–0.89)
1–4	1.05 (0.76–1.46)	1.16 (0.82–1.63)
>5	1.25 (0.93–1.67)	1.35 (0.99–1.83)

Multivariate analysis: adjusted for patient’s age, sex, type of medical security, admission route, OP type, lymph node dissection, hospital location, and hospital type.

**Table 4:** Comparison of Charlson and Elixhauser comorbidities with respect to nursing indicators.

Nursing Indicator	Total Number	Number of Events	Incidence Rate (%)	Model													
				Base Model		Using Item				Using Continuous Variable				Using Category			
						Base Model + Charlson Items		Base Model + Elixhauser Items		Base Model + Charlson Score		Base Model + Elixhauser Score		Base Model + Charlson Score Level		Base Model + Elixhauser Score Level	
				AIC	C-Stat	AIC	C-Stat	AIC	C-Stat	AIC	C-Stat	AIC	C-Stat	AIC	C-Stat	AIC	C-Stat
NSO	33,323	3545	10.64	20,751.12	0.716	20,584.48	0.726	20,518.49	0.729	20,591.04	0.725	20,596.00	0.725	20,648.15	0.722	20,583.52	0.725
In-hospital mortality	33,323	130	0.39	1486.75	0.824	1476.83	0.853	1488.23	0.863	1465.19	0.847	1470.52	0.839	1475.91	0.833	1466.20	0.852
CNS complications	33,322	187	0.56	2140.44	0.775	2140.20	0.794	2128.32	0.804	2124.45	0.785	2113.61	0.788	2129.92	0.786	2112.74	0.803
Shock/Cardiac arrest	33,306	944	2.83	7724.43	0.769	7709.42	0.776	7713.34	0.779	7706.26	0.773	7695.83	0.774	7709.74	0.771	7689.43	0.775
Hospital- acquired pneumonia	31,692	545	1.72	5144.90	0.739	5130.53	0.747	5461.91	0.718	5120.89	0.743	5118.75	0.744	5130.09	0.743	5132.94	0.742
Upper gastrointestinal tract bleeding	33,292	547	1.64	4786.23	0.769	4753.04	0.793	5288.10	0.784	4768.17	0.781	4775.45	0.776	4774.01	0.776	4771.57	0.781
Pressure ulcer	33,317	224	0.67	2543.25	0.734	2527.21	0.759	2541.28	0.757	2513.92	0.753	2520.53	0.749	2521.56	0.746	2507.10	0.750
Deep vein thrombosis	33,323	236	0.71	2694.39	0.695	2680.62	0.732	2539.31	0.741	2668.50	0.721	2667.87	0.722	2672.14	0.714	2659.68	0.723
Urinary tract infection	33,292	175	0.53	2088.11	0.725	2064.348	0.740	2078.35	0.746	2063.31	0.729	2077.75	0.726	2070.20	0.727	2074.25	0.724
Wound infection	33,323	267	0.80	2846.23	0.788	2831.57	0.797	2849.95	0.799	2829.26	0.793	2835.25	0.789	2838.86	0.791	2840.61	0.789
Pulmonary failure	33,323	76	0.23	1012.61	0.751	1031.60	0.771	1042.45	0.785	1014.55	0.748	1014.57	0.752	1013.88	0.739	1017.19	0.731
Physiologic/ metabolic derangement	33,306	1157	3.47	9468.18	0.719	9445.96	0.725	9405.67	0.733	9437.82	0.723	9435.62	0.723	9451.06	0.722	9432.31	0.722

NSO: nursing-sensitive outcomes; CNS complications: central nervous system complications; AIC: the Akaike information criterion; C-stat: C-statistics; base model: adjusted for patient’s age, sex, type of medical security, admission route, OP type, lymph node dissection, hospital location, and hospital type.

## Data Availability

Authors analyzed the data within the database system of the Korea Health Insurance Review and Assessment Service (a government agency), which cannot be transferred to outside the agency.
